# Reliability, Validity, and Measurement Invariance of the General Anxiety Disorder Scale Among Chinese Medical University Students

**DOI:** 10.3389/fpsyt.2021.648755

**Published:** 2021-05-19

**Authors:** Chi Zhang, Tingting Wang, Ping Zeng, Minghao Zhao, Guifang Zhang, Shuo Zhai, Lingbing Meng, Yuanyuan Wang, Deping Liu

**Affiliations:** ^1^The Key Laboratory of Geriatrics, Beijing Institute of Geriatrics, Beijing Hospital, National Center of Gerontology, National Health Commission, Beijing, China; ^2^Institute of Geriatrics Medicine, Chinese Academy of Medical Sciences, Beijing, China; ^3^International Student Office of International Cooperation Department, Peking University Health Science Center, Beijing, China; ^4^School of Basic Medicine, Peking University Health Science Center, Beijing, China; ^5^Department of Education, Beijing Hospital, National Center of Gerontology, Beijing, China; ^6^Department of Cardiology, Beijing Hospital, National Center of Gerontology, Beijing, China; ^7^National Center for Health Professions Education Development, Peking University Health Science Center, Beijing, China

**Keywords:** general anxiety disorder scale, medical students, classical test theory, item response theory, measurement invariance

## Abstract

**Background:** Medical students are affected by high levels of general anxiety disorder. However, few studies have specifically focused on the applicability of universal anxiety screening tools in this sample. This study was aimed to evaluate the psychometric property of the 7-item Generalized Anxiety Disorder Scale (GAD-7) among Chinese medical university students.

**Methods:** A questionnaire survey was conducted among 1,021 medical postgraduates from six polyclinic hospitals. Internal consistency and convergent validity of the GAD-7 were evaluated. Factor analyses were used to test the construct validity of the scale. An item response theory (IRT) framework was used to estimate the parameters of each item. Multi-group confirmatory analyses and differential item function analyses were used to evaluate the measurement equivalence of the GAD-7 across age, gender, educational status, and residence.

**Results:** Cronbach's α coefficient was 0.93 and the intraclass correlation coefficients ranged from 0.71 to 0.87. The GAD-7 summed score was significantly correlated with measures of depression symptoms, perceived stress, sleep disorders, and life satisfaction. Parallel analysis and confirmatory factor analysis supported the one-factor structure of the GAD-7. Seven items showed appropriate discrimination and difficulty parameters. The GAD-7 showed good measurement equivalence across demographic characteristics. The total test information of the scale was 22.85, but the test information within the range of mild symptoms was relatively low.

**Conclusions:** The GAD-7 has good reliability, validity, and measurement invariance among Chinese medical postgraduate students, but its measurement precision for mild anxiety symptoms is insufficient.

## Introduction

The prevalence of mental health disorders has increased considerably among medical students including postgraduates (1). These students are affected by higher levels of anxiety than students who major in other disciplines (2–5) as well as the general population (6, 7). Anxiety has garnered little attention and is often undetected or undertreated in the general population. In particular, only a small number of college students undergo timely screening (8). Generalized anxiety disorder (GAD) is the most common form of anxiety, which is characterized by excessive and persistent worry (9, 10). Studies have shown that GAD was correlated with academic performance (11), depression symptoms (12, 13), sleep problems (14), and adverse events (15).

Several systematic reviews have described high levels of general anxiety disorder among medical students in the US (16), Canada (3), Brazil (17), and China (18). Anxiety is most prevalent among medical students from the Middle East and Asian countries (19). A recent review including 10 investigation studies showed that the prevalence of anxiety among Chinese medical students is 21%, which is higher than that of students majoring in other subjects, as well as medical students from other Asian countries (20). A cross sectional study showed that 11% of postgraduate medical residents in Bangladesh had anxiety disorders (21). Medical university students are affected by various sources of stress, such as academia, employment, family, tutors, and a harsh health service environment. Although researchers are concerned about the prevalence of anxiety disorder among medical students, more attention should be paid to the early screening and a valid tool for GAD screening needs to be generally accepted in this sample. However, the literature regarding this specific population has been relatively insufficient.

The 7-item Generalized Anxiety Disorder Scale (GAD-7) (9), recommended by the Diagnostic and Statistical Manual of Mental Disorders, Fourth Edition (DSM-IV) (22), is a common instrument used in the screening of generalized anxiety disorders because of its simplicity and operability. The GAD-7 has been translated into different languages, including Chinese, over the last two decades (23–26). The reliability, validity, and diagnostic capability of the GAD-7 have been confirmed, but the majority of previous psychometric studies focused on clinical settings rather than general populations (12, 23, 27–29). Although the GAD-7 has been widely used for anxiety screening among medical students (30–32), few studies have systematically evaluated its measurement properties in this sample. Besides, previous studies have focused on the psychometric performance of the overall scale, but little attention has been paid to the characteristics or measurement invariance of individual items. The measurement equivalence is an important attribute of a screening instrument, as it ensures the comparability of measurement values across different subsamples. Therefore, it is necessary to evaluate the GAD-7 comprehensively with methodologies that combine classical test theory (CTT) and item response theory (IRT). The IRT framework test the probability of subjects' response according to particular models and then evaluates parameters of the measurement tool. These methods were originally designed to evaluate examination tools and are recently widely used to assess the suitability of health-related scales (33, 34).

This study was designed to evaluate the reliability, validity, and measurement invariance of the GAD-7 using a sample of medical university students. We also aimed to provide reasonable suggestions of its application in practice.

## Materials and Methods

### Participants

The study participants were 1,021 full-time medical postgraduates from six polyclinic hospitals of Peking University Medical College or Peking Union Medical College. These hospitals were Beijing Hospital, the First Hospital of Peking University, the People's Hospital of Peking University, the Third Hospital of Peking University, Peking Union Medical College Hospital, and the Cancer Hospital of The Chinese Academy of Medical Sciences. In each hospital, more than 50 percent of the total postgraduate students were selected during the survey. We estimated sample size on the basis of factor analysis and item sample ratio method. As some researchers recommend, a sample of 300–1,000 in factor analysis is excellent (35) and a sample item ratio between 10 and 20 indicates sufficient (36). Respondents in the current study included 630 (61.71%) master's and 391 (38.29%) doctoral medical students.

### Procedures and Ethic

A cross-sectional questionnaire survey was conducted from April to June, 2020, and the management staff of each hospital collected the data. The ethics committee of Beijing Hospital approved this study (2020BJYYEC-231-01). All respondents gave informed consent and volunteered to participate in the study. The background and purpose of the survey, as well as informed consent, were explained on the first page of the questionnaire. In order to ensure an effective recovery rate, each respondent could receive a feedback report by email after submission. A total of 1,108 questionnaires were collected and 87 invalid questionnaires (either incomplete within the allotted time, had unidentifiable information, or too many repetitive responses) were excluded. Thus, 1,021 valid questionnaires were obtained and included in the analysis.

### Measurements

General anxiety disorders were measured with a Chinese GAD-7 version. The Chinese GAD-7 version was firstly translated by He and colleagues using standard translation methods in 2010 (37). In the current study, we quoted the Chinese GAD-7 version from He's study and further translated it back into English by a doctor of psychiatry, two specialists in medical education, and one English native overseas postgraduate. These cross-cultural adaptation procedures ensured the semantic equivalence between the translated version and the original. Besides, as the statements of questions in the GAD-7 are relatively concise, university students could understand the meaning easily, thus the face validity and content validity of the Chinese GAD-7 version were supported. Previous studies have demonstrated the GAD-7's appropriate screening utility in clinical samples and the general population (23, 27, 38, 39). Its unidimensional structure has been demonstrated in many published studies employing different methodologies (27, 29), with a few exceptions (12, 40). The GAD-7 is a 7-item self-report measurement designed to screen the presence of general anxiety disorders over the previous 2 weeks (9). Items consist of seven statements about worry or somatic tension, which are rated on a four-point Likert scale as follows: 0 (not at all); 1 (several days); 2 (more than half the number of days); and 3 (nearly every day), indicating frequency levels of GAD symptoms. The GAD-7 summed score ranges from 0 to 21, with cutoff points of 5, 10, and 15 allowing researchers to classify the anxiety as none/normal (0–4), mild (5–9), moderate (10–14), and severe (15–21) (41). However, the cutoff score for the prevalence of general anxiety disorders has not been consistent among multiple samples. The original validation study of the GAD-7 in the primary care setting, adopted 9/10 as the cutoff score (9), while the recommended cutoff scores range from 7 to 13 for different versions (23, 26, 27, 41–44). Furthermore, a small number of studies have used a 4/5 score as an optimal cutoff (45).

The Patient Health Questionnaire 9-item depression scale (PHQ-9) (46), a valid self-administered depression screening and diagnostic tool, was used to measure depression symptoms. The Cronbach's α coefficient of PHQ-9 in this study was 0.89. The 10-item Perceived Stress Scale (PSS-10) (47) was used to measure perceived stress. The PSS-10 is one of the most frequently used self-report psychological questionnaires, which is widely used across various cultures and populations (48). It showed appropriate consistency reliability with an α coefficient of 0.91. The Athens Insomnia Scale (AIS) (49) was used to quantify the presence of insomnia among study participants. The AIS is widely used in the general population and included eight three-point Likert items. It showed appropriate consistency reliability with an α coefficient of 0.87.

### Statistical Analysis

Continuous variables were described as the mean ± standard deviation (mean ± SD), and categorical variables were described as numbers with percentages [*n* (%)]. The Student's *t* test or Wilcoxon rank sum test was used to compare the differences of GAD-7 scores among different groups. Spearman correlation coefficient was used to analyze the correlation between GAD-7 score and other measured outcomes. Statistical significance was accepted at the two-sided 0.05. Internal consistency of the scale was evaluated using the Cronbach's α coefficient and Guttmann's coefficient, with an α coefficient >0.7 indicating good internal reliability (50). An exploratory factor analysis (EFA) using the principal component method was performed to explore the factor structure. Parallel analyses (PA) (51) were used to retain factors with 500 random data matrices. The retained eigenvalues should meet the K1 criterion (≥1) and greater than the average or the 95th percentile of the random samples. Factor loading >0.6 in exploratory factor analysis (EFA) is considered acceptable. Confirmatory factor analyses (CFA) with robust weighted least squares estimation were conducted in Mplus (version 7.4) in cases of violation of the multivariate normality assumption. χ^2^/*df* , root mean square error of approximation (RMSEA), comparative fit index (CFI), and normed fit index (NFI) were used to evaluate the fitness. The model is considered to have a good fit with a χ^2^/*df* of 5 or less, a RMSEA of 0.1, a CFI and NFI >0.90 (52).

The IRT analysis with a fitted Semejima graded response model was implemented to estimate the discrimination (a) and difficulty (b) parameters of seven items using the IRTPRO version 4.2 software. Prior to the implementation of the IRT, the unidimensionality assumption was tested using factor analyses. The local independence was confirmed using the χ^2^LD statistic (53) and residuals covariance (54). A χ^2^LD statistic <10 and a standardized residual covariance <0.2 between two items indicated an acceptable level of local independence (54). The item characteristic curve (ICC) was used to establish the relationship between subjects' potential trait and their responses, and the item information curve (IIC) was used to evaluate the measurement precision through the test information function (TIF). The measurement precision of a scale is sufficient when the total test information is above 16 (55). In addition, by applying the posterior estimation method, the transformation relationship between the original sum score and IRT characteristic score was established (56).

Factorial invariance of the GAD-7 across age, gender, education, and residence was tested by a multigroup confirmatory factor analysis approach, which consisted of a series of nested confirmatory steps (57). Configural invariance (free parameters), metric invariance (constraints of equivalent factor loadings), scalar invariance (further constraints of the intercepts), and strict invariance (further constraints of residual variances) models were tested across subgroups. A no-significant _Δ_χ^2^ (*P* > 0.05); a _Δ_CFI value <0.01; and a _Δ_RMSEA value <0.15 were used to compare the fit of nested models (58). We examined measurement invariance of item parameters using differential item functioning (DIF) methods (59). The DIF occurred when the relationship between the latent variable and item responses differed on item parameters across subgroups. The existence of the DIF suggests that the differences between groups may not be due to actual differences between groups in the survey variables, but to other factors, such as the measurement tool itself or unknown external factors (60). A no-significant _Δ_χ^2^ (*P* > 0.05) at specific degrees of freedom indicated acceptable parameter invariance (60).

## Results

### Demographic Characteristics

A total of 1,021 postgraduate students (26.01 ± 2.46 years) completed the survey, including 61.71% master and 38.29% doctoral students. The majority were female (65.36%) and clinical medical students (76.64%). The average GAD-7 score was 6.29 ± 3.58 and the distribution of points for each item was described in Table 1. Among the participants, 34.28% (350/1,021) had no anxiety; 49.07% (501/1,021) had mild anxiety; 12.34% (126/1,021) had moderate anxiety; and 4.31% (44/1,021) had severe anxiety. The socio-demographic characteristics according to the GAD-7 scores of the postgraduate students were presented in Table 2.

**Table 1:** Distributions of scores of 7 items in the General Anxiety Disorder Scale [*n* (%)].

**Contents**	**Not at all**	**Several days**	**More than half the number of days**	**Nearly every day**	**Mean**	**SD**
1. Feeling nervous, anxious, or on edge	134 (13.12)	718 (70.32)	128 (12.54)	41 (4.02)	1.07	0.64
2. Becoming easily annoyed or irritable	217 (21.25)	642 (62.88)	136 (13.32)	26 (2.55)	0.92	0.67
3. Feeling afraid, as if something awful might happen	366 (35.85)	509 (49.85)	117 (11.46)	29 (2.84)	0.81	0.73
4. Worrying too much about different things	370 (36.24)	485 (47.50)	133 (13.03)	33 (3.23)	0.83	0.77
5. Being so restless that it is hard to sit still	297 (29.09)	579 (56.71)	119 (11.66)	26 (2.55)	0.88	0.71
6. Not being able to stop or control worrying	321 (31.44)	547 (53.57)	127 (12.44)	26 (2.55)	0.86	0.72
7. Trouble relaxing	345 (33.79)	506 (49.56)	136 (13.32)	34 (3.33)	0.86	0.77

*SD, standard deviation*.

**Table 2:** Characteristics and summed GAD-7 score of 1,021 medical students.

**Characteristics**		**N**	**Proportion (%)**	**Mean**	**SD**	***P*-value**
Age	19–25	461	45.15	6.53	3.51	0.126^†^
	26–37	560	54.85	6.09	3.42	
Gender	Male	356	34.87	6.38	3.34	0.594^†^
	Female	668	65.36	6.28	3.71	
Education	Doctor	391	38.29	6.25	3.41	0.108^†^
	Master	630	61.71	6.24	3.71	
Degree	Academic	461	44.93	6.04	3.68	0.063^§^
	Professional	563	54.82	6.54	3.43	
Residence	Rural	354	34.30	6.55	3.29	0.178^†^
	City or town	670	64.86	6.19	3.68	
Income	Poverty^*^	527	50.97	6.87	3.63	0.002^§^
	No-poverty	497	48.02	5.95	3.48	
Major	Clinical medicine	794	76.64	6.11	3.54	0.006^§^
	Others	230	22.18	7.03	3.89	
Institutional satisfaction	Yes	887	85.29	5.98	3.61	<0.001^§^
	No	137	13.16	8.51	3.01	
Major satisfaction	Yes	837	80.33	5.97	3.57	<0.001^§^
	No	187	17.93	7.86	3.31	
Relationship with tutors	Good	867	83.05	6.04	3.54	<0.001^§^
	Bad	157	15.02	7.85	3.61	
Exercise habits	Yes	397	37.95	5.55	3.62	<0.001^§^
	No	627	59.89	6.81	3.50	

* *The annual household income is <100,000 yuan*.

† *Student's t test*.

§ *Wilcoxon rank-sum test*.

*SD, standard deviation*.

### Reliability, Validity, and Factor Structure of the GAD-7

**Figure 1:**
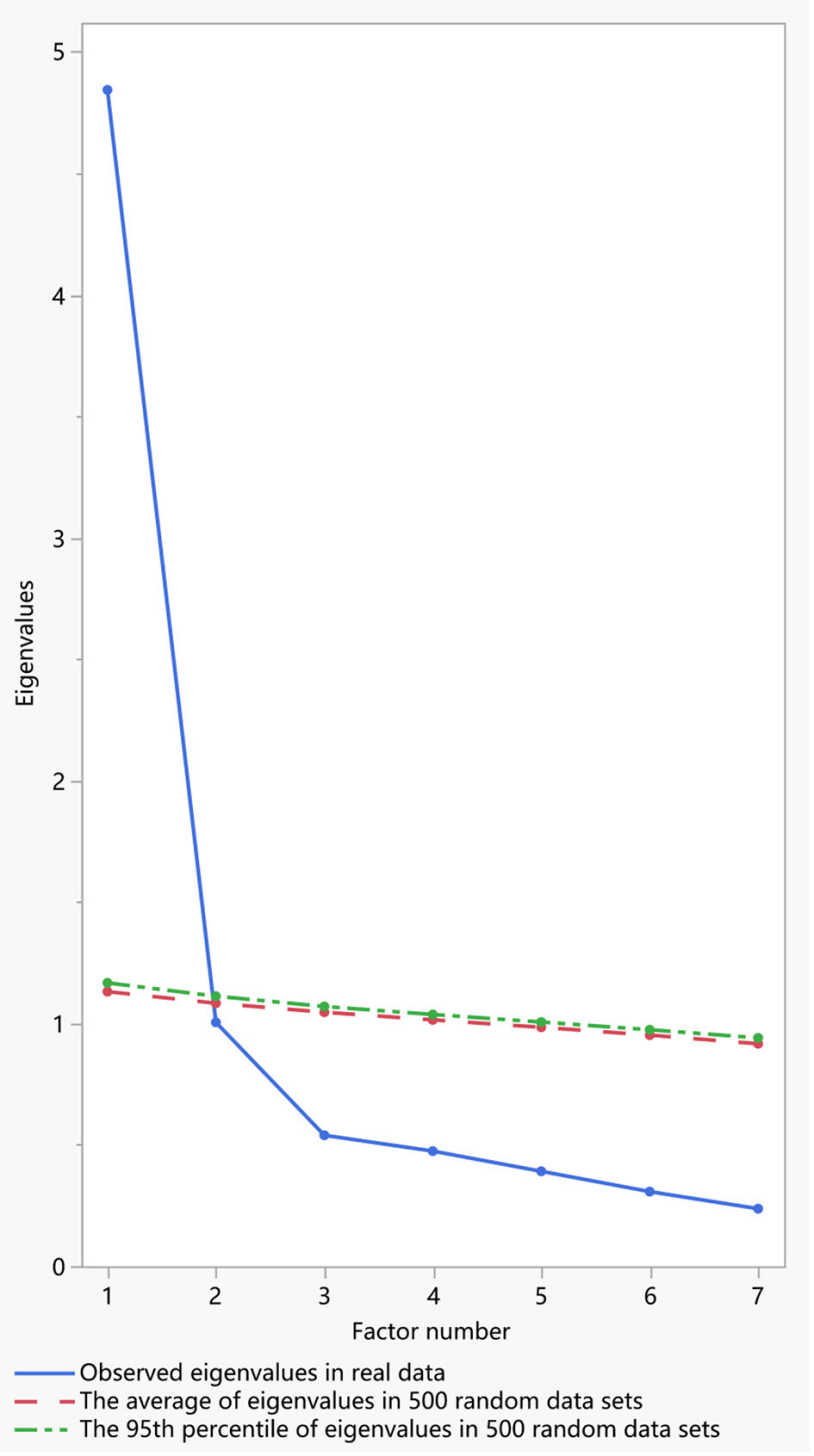
Scree plot of the 7-item Generalized Anxiety Disorder Scale in parallel analysis.

**Figure 2:**
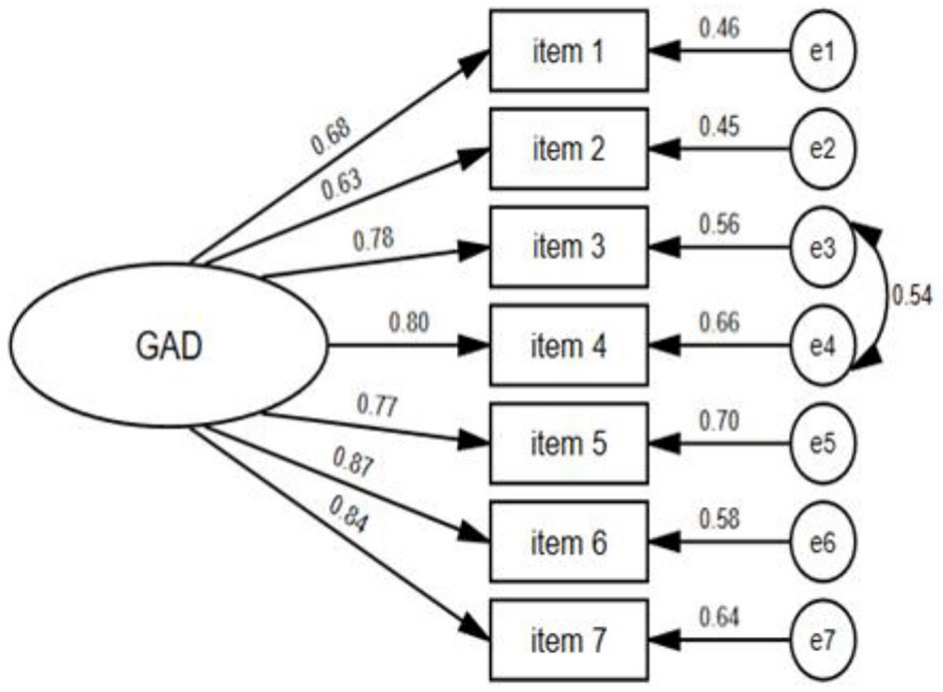
Unidimensional confirmatory factor analysis model of the 7-item Generalized Anxiety Disorder Scale.

The overall α coefficient of the GAD-7 was 0.93 and the Guttmann's coefficient was 0.89. The summed GAD-7 score was statistically significant correlated with scores of the PHQ-9 (*r* = 0.78, *P* < 0.001), PSS-10 (*r* = 0.71, *P* < 0.001), AIS-8 (*r* = 0.67, *P* < 0.001), and SWLS-5 (*r* = −0.38, *P* < 0.001). As Table 2 showed, the α coefficient of the scale was reduced when a specific item was removed. The intraclass correlation coefficients between scores of seven items and the summed GAD-7 score ranged from 0.71–0.87 (*P* < 0.001). The KMO statistic was 0.92 and the significance of Bartlett's test of sphericity (χ^2^ = 4,997.63, *df* = 21, *P* < 0.001) indicated that the data was suitable for factor extraction. A parallel analysis employing the principal component method was used to determine the number of factors, and one common factor was extracted. The eigenvalue of this factor was 4.82 accounting for 66.02% of the variation and the scree plot was showed in Figure 1. As Table 3 showed, the loadings of seven items on this factor were >0.7. A CFA with weighted least square estimation was used to test the one-factor structure of the GAD-7. The modification index between item 3 and item 4 was 83.52, and the CFA model was modified by establishing the residual covariation correlation between the two items. The adaptability of the modified model was then significantly improved (χ^2^/*df* = 3.48, CFI = 0.97, NFI = 0.96, RMSEA = 0.05) and the factor loading of each item in the CFA model was >0.6. This indicated that the unidimensional structure showed excellent suitability to the data. The CFA model of the GAD-7 is shown in Figure 2.

**Table 3:** Item analyses of the GAD-7 based on classical test theory and item response theory.

**Contents**	**CTT**				**IRT**				
	α coefficient^*^	**ICC**	**EFA loading**	**CFA loading**	**a (*S.E*.)^*†*^**	**b1 (*S.E*.)^*‡*^**	**b2 (*S.E*.)^§^**	**b3 (*S.E*.)^¶^**	**Information (θ)**
1. Feeling nervous, anxious, or on edge	0.91	0.74	0.74	0.68	2.54 (0.26)	−1.22 (0.11)	1.69 (0.12)	2.60 (0.19)	2.49 (1.23)
2. Becoming easily annoyed or irritable	0.91	0.71	0.70	0.63	1.90 (0.17)	−0.77 (0.10)	1.87 (0.14)	3.19 (0.28)	1.16 (1.32)
3. Feeling afraid, as if something awful might happen	0.89	0.85	0.85	0.78	4.25 (0.46)	−0.18 (0.07)	1.52 (0.09)	2.58 (0.18)	3.42 (1.24)
4. Worrying too much about different things	0.89	0.86	0.86	0.80	4.79 (0.57)	−0.18 (0.07)	1.46 (0.09)	2.40 (0.15)	4.10 (2.14)
5. Being so restless that it is hard to sit still	0.90	0.79	0.79	0.77	2.68 (0.24)	−0.40 (0.08)	1.84 (0.12)	2.91 (0.23)	2.70 (1.36)
6. Not being able to stop or control worrying	0.89	0.87	0.87	0.87	4.73 (0.70)	−0.30 (0.07)	1.61 (0.09)	2.55 (0.17)	5.41 (1.15)
7. Trouble relaxing	0.90	0.84	0.84	0.84	3.51 (0.35)	−0.23 (0.07)	1.68 (0.10)	2.48 (0.17)	3.01 (1.11)

* *Cronbach's α coefficients of GAD-7 when the specific item was deleted*.

† *Discrimination parameters of items*.

‡ *Difficulty parameters of items for response category 1 (several days)*.

§ *Difficulty parameters of items for response category 2 (more than half the days)*.

¶ *Difficulty parameters of items for response category 3 (nearly every day)*.

*GAD-7, 7-item Generalized Anxiety Disorder Scale; CTT, classical test theory; IRT, item response theory; ICC, intraclass correlation coefficient; EFA, exploratory factor analysis; CFA, confirmatory factor analysis; S.E., standard error*.

We then tested the factorial invariance using the multi-group confirmatory factor analysis (MGCFA) framework. The configural invariance model was used as a basic model and three restrictive models were tested step by step. As summarized in Table 4, the metric invariance model and scalar invariance model showed excellent fitness across age, age, gender, education, and residence (*P* > 0.05, ΔCFI <0.01). The strict invariance model only showed acceptable fitness across residence (*P* = 0.236, ΔCFI = 0.004).

**Table 4:** Factorial invariance analyses of the 7-item Generalized Anxiety Disorder Scale across age, gender, education, and residence.

**Models**	**Metric invariance model**					**Scalar invariance model**					**Strict invariance model**				
**Index**	**Δχ^2^**	***df***	***P***	**ΔCFI**	**_**Δ**_RMSEA**	**Δχ^2^**	***df***	***P***	**ΔCFI**	**_**Δ**_RMSEA**	**Δχ^2^**	***df***	***P***	**ΔCFI**	**_**Δ**_RMSEA**
Age	11.4	6	0.076	0.002	0.002	11.4	7	0.121	0.002	0.003	73.9	15	0.000	0.015	0.214
Gender	8.23	6	0.222	0.002	0.003	8.27	7	0.309	0.002	0.004	50.4	15	0.000	0.010	0.185
Education	8.44	6	0.208	0.002	0.005	11.4	7	0.120	0.002	0.003	48.0	15	0.000	0.009	0.179
Residence	5.68	6	0.460	0.001	0.004	12.5	7	0.085	0.002	0.004	18.5	15	0.236	0.004	0.005

*CFI, comparative fit index; RMSEA, root mean square error of approximation*.

### Item Characteristics of the GAD-7

**Figure 3:**
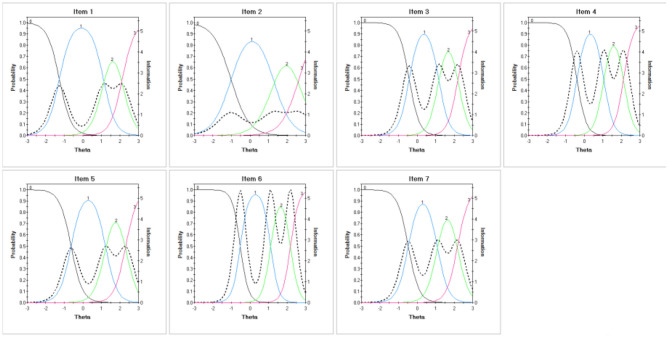
Item characteristic curves and item information curves of seven items in the GAD-7.

**Figure 4:**
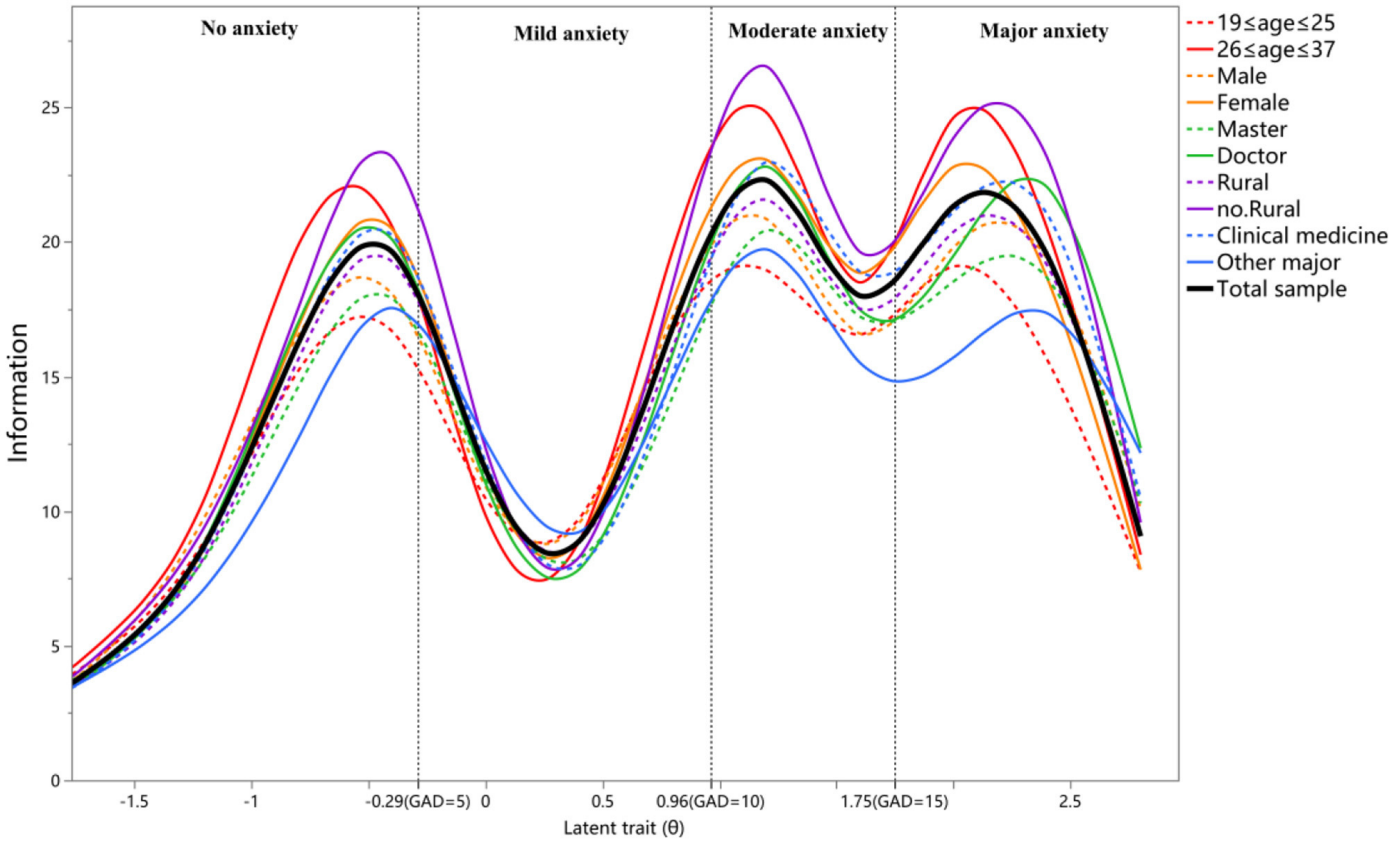
Test information function curves of the GAD-7 according to multiple subgroups.

As the results of EFA and CFA supported the unidimensional structure of the GAD-7, we further conducted the χ^2^LD statistic matrix (Supplementary Table 1) and residual covariance matrix (Supplementary Table 2) between any two items to test its local independence. The χ^2^LD statistics were all <10 (0.42 to 9.30) and the residual covariances were <0.2 (−0.007 to 0.053), which indicated an appropriate local independence feature of the GAD-7. Among the two matrices, items 3 and 4 showed the highest χ^2^LD statistic (9.3) and highest residual covariance (0.053). An item response analysis with a Semejima graded model was used to estimate the parameters of seven items. The discrimination parameter of seven items ranged from 1.90 to 4.79, and the difficulty parameter ranged from −1.22 to 3.19 with a monotonically increasing trend. All seven items had sufficient test information with a corresponding local trait (θ). We summarized the ICC and IIC of seven items in Figure 3 and parameter values are shown in Table 3. We listed the conversion between the original GAD summed scores and the IRT trait scores (Supplementary Table 3), and divided the horizontal coordinate of the test information curve of the GAD-7 into four anxiety category levels. As Figure 4 shows, the total test information of the GAD-7 among medical postgraduate students was 22.85, and the corresponding latent trait level located at 1.38. However, the test information within the range of mild anxiety symptoms (5 ≤ GAD score <10) was relatively low.

We further analyzed the differential item function of each item across four subgroups. The results of parameter invariance are summarized in Table 5. No statistically significant differences were found in either discrimination or difficulty parameters (*P* > 0.05) according to the _Δ_χ^2^, which indicated excellent equivalence for the seven items. Furthermore, the test information function curves of the different subgroups were close to the curve of the total sample (Figure 4). These curvilinear paths further supported the measurement invariance of the GAD-7.

**Table 5:** Measurement invariance of item parameters of the GAD-7 across subgroups.

**Subgroups**	**Item**	Total_Δ_χ^2^^*^	***df***	***P***	**$\Delta \chi_{\text{a}}^{2}$^†^**	***df***	***p***	**_Δ_χ^2^b^§^**	***df***	***p*-value**
Age	1	4.2	4	0.386	1.4	1	0.233	2.7	3	0.437
	2	2.7	4	0.607	2.0	1	0.158	0.7	3	0.869
	3	4.6	4	0.327	1.3	1	0.260	3.4	3	0.339
	4	0.8	4	0.933	0.1	1	0.808	0.8	3	0.855
	5	5.3	4	0.262	2.5	1	0.134	0.8	3	0.851
	6	5.7	4	0.227	2.7	1	0.130	1.0	3	0.811
	7	2.1	4	0.724	0.7	1	0.397	1.3	3	0.719
Gender	1	4.5	4	0.338	0.8	1	0.361	3.7	3	0.296
	2	1.7	4	0.799	0.4	1	0.526	1.3	3	0.741
	3	0.4	4	0.979	0.2	1	0.648	0.2	3	0.972
	4	1.5	4	0.830	0.2	1	0.679	1.3	3	0.726
	5	2.1	4	0.716	0.0	1	0.915	2.1	3	0.552
	6	1.5	4	0.828	0.5	1	0.490	1.0	3	0.798
	7	2.8	4	0.587	0.7	1	0.414	2.2	3	0.540
Education	1	4.4	4	0.360	0.3	1	0.610	4.1	3	0.252
	2	2.8	4	0.593	0.1	1	0.726	2.7	3	0.445
	3	3.4	4	0.489	0.2	1	0.637	3.2	3	0.362
	4	4.1	4	0.391	0.0	1	0.907	4.1	3	0.251
	5	0.5	4	0.976	0.0	1	0.909	0.5	3	0.926
	6	0.5	4	0.970	0.0	1	0.958	0.5	3	0.912
	7	7.0	4	0.134	3.6	1	0.091	2.4	3	0.495
Residence	1	4.5	4	0.348	1.2	1	0.271	3.3	3	0.356
	2	3.4	4	0.497	2.1	1	0.145	1.3	3	0.741
	3	4.6	4	0.337	3.1	1	0.077	1.4	3	0.696
	4	5.8	4	0.218	2.7	1	0.101	3.1	3	0.381
	5	2.7	4	0.608	0.3	1	0.616	2.5	3	0.484
	6	1.1	4	0.896	0.8	1	0.387	0.3	3	0.953
	7	2.3	4	0.672	0.3	1	0.568	2.0	3	0.569

* *Δχ^2^ statistic for the specific item*.

† *Δχ^2^ statistic for discrimination parameter(a) of the specific item*.

§ *Δχ^2^ statistic for difficulty parameter(b) of the specific item*.

## Discussion

As far as the authors know, this is the first study to evaluate the psychometric properties of the GAD-7 among medical university students combining CTT and IRT. We observed a higher prevalence of general anxiety disorders (65.72%) than previous reports in China (18). This indicated that psychological impairment was a common problem among Chinese medical students. In addition, the high incidence could also be attributed to the influence of COVID-19, as teaching tasks in universities of Beijing had not fully recovered during the survey period. Notably, the differences observed in the anxiety detection rate was related to the selection of the GAD-7's cutoff (16). When the threshold of 9/10 was applied, the detection rate of general anxiety disorder dropped to 16.65%. The results of IRT analysis showed that the GAD-7 had considerably lower test information for subjects with mild anxiety symptoms. This innovative finding supported the importance of careful selection of cutoff values in clinical practice, and the necessity of clinical diagnosis in subjects with mild symptoms (GAD scores ranging from 5 to 10). No significant differences were found in the subjects' GAD scores across different age, gender, education status, or residence subgroups. This indicated weak associations between general anxiety and demographic characteristic among the medical university students. The GAD scores were closely related to family income and satisfaction (with college, major, or tutor), and were consistent with the results of previous studies of medical students (61). The above results show that negative emotions among medical students are an important potential risk factor of general anxiety disorders.

In the current study, we implemented standardized back-translation and cross-cultural adaptation procedures to ensure the content validity of the Chinese GAD-7 version (36). The GAD-7 had a good internal consistency reliability coefficient of 0.93, which is consistent with that reported in previous studies ranging from 0.74 (42) to 0.94 (25). The strong correlation coefficient between the GAD-7 and PHQ-9 (*r* = 0.78) has also been observed among other samples (12, 13, 62). These findings suggest that anxiety disorders frequently occur alongside depression symptoms. Several previous studies have also confirmed the association between the GAD-7 and factors such as stress (12), sleep disorders (28), and life satisfaction (28). Significant correlations between the GAD-7 and theoretically related measurements support the scale's convergent validity and discrimination ability for subjects with differences in psychological status. These results are consistent with those of previous studies of multiple populations (13, 27, 41, 63). Although the one-dimensional structure of the GAD-7 proposed by its developer is not consistent across all studies, the construct validity of its unidimensional structure was confirmed in the current study. This finding was consistent with the majority of published studies conducted in both the primary care setting and the general population (9, 25, 63). Nevertheless, a two-factor structure was reported by Satomi (40) among Japanese adult populations, as well as Kertz's (12) study in an acute psychiatric sample. Heterogeneity of the sample and differences in methodology may explain these conflicting results. We modified the CFA model by establishing a residual correlation between item 3 (“Feeling afraid as if something awful might happen”) and 4 (“Worrying too much about different things”). The residual pair between specific items is a common method used to improve the scale's fitness, and has been applied in previous studies among Portuguese college students (13), American outpatients (64), and heterogeneous psychiatric populations (65). There was some overlap between content of item 3 and 4. Furthermore, the LDχ^2^ statistic between the two items (9.3) was higher than that of other pairs. This indicated that these two questions reflected an ambiguous trait, other than general anxiety (e.g., fear). When we removed either of the two items, the total test information of the GAD-7 was significantly reduced. Thus, we recommend retaining all items for specific applications. The metric and scalar invariance models of the unidimensional GAD-7 showed excellent equivalence across subgroups, which has also been confirmed in various clinical and general population studies (12, 13, 39, 64). However, the strict invariance model was not equivalent across demographic characteristics. This might be related to the heterogeneity of residual covariance between different items among subgroups.

The GAD-7 has good local independence among medical university students. This is an important characteristic of an ideal scale and one of the preconditions for IRT analysis that is often ignored by researchers (27). The difficulty and discrimination parameters of seven items were within an appropriate range (Table 3) and the total test information of the scale was relatively high (22.85). These findings are consistent with those of Zhong's study in pregnant women (27). Although some psychological experts suggested collapsing the response categories, “more than half the days” and “nearly every day” in a graded response scale, owing to potentially disordered thresholds (66, 67), the difficulty parameters of the four response categories increased monotonically in the present results. This indicated that response categories were used in a reasonable and ordered manner. According to the summarized ICC (Figure 3), the curves of the four response categories 0–4 were significantly spaced, which is inconsistent with the findings of another study of antepartum women in two low-income countries (68). The seven items showed appropriate measurements in DIF analyses (Table 5). This indicated that the GAD-7 was fair among the subsamples, which is consistent with the findings of Pascal's study among primary care patients (69). Besides, we used the test information curves (Figure 4) to describe the measurement precision of the GAD-7, according to different screening outcomes, which is helpful in choosing optimal cutoff scores. One novelty of the present study is the fact that the GAD-7 had a lower precision for persons with mild anxiety symptoms (with scores ranging from 5–10). Barthel also confirmed that the GAD-7 items measured well at higher anxiety levels, but not as well at lower levels (68). These findings strengthen the necessity for the clinical diagnosis of persons with mild anxiety symptoms and rigorous exposition of cutoff scores in practical applications. The GAD-7 had relatively sufficient test information within the range of non-anxiety symptoms, as well as moderate and major symptoms, indicating that it is a valid screening tool for the sample. We further constructed the IIC (Figure 4) across different demographic characteristics, and the basic shapes of all curves were relatively similar. Moreover, the test information curves among subgroups showed very little fluctuation around the curve of the total sample, which also supported its measurement equivalence.

This study had some limitations. Firstly, we did not confirm the inter-intra rater reliability and concurrent validity of the Chinese GAD-7 version. Secondly, the optimal cutoff was not identified owing to the lack of clinical diagnoses. Thirdly, the sample used in this study originated from only one city in China, and extrapolation to other populations needs to be further verified. In the future study, we will test the screening ability of specific anxiety scales in conjunction with clinical diagnosis, as well as expand the scope of random sampling nationwide.

## Conclusion

The 7-item General Anxiety Disorder Scale showed acceptable reliability, validity, and measurement invariance among Chinese medical postgraduates. The optimal cutoff score of the GAD-7 should be considered with caution, because of its insufficient measurement precision for symptoms of mild anxiety.

## Data Availability Statement

The original contributions generated for this study are included in the article/Supplementary Material, further inquiries can be directed to the corresponding author/s.

## Ethics Statement

The studies involving human participants were reviewed and approved by the ethics committee of Beijing Hospital approved this study (2020BJYYEC-231-01). The patients/participants provided their written informed consent to participate in this study.

## Author Contributions

CZ, TW, and DL proposed the concept and design. CZ analyzed and interpreted the data, wrote the manuscript. PZ, MZ, GZ, LM, YW, and SZ drafted and edited the manuscript. CZ and DL supervised the study and obtained funding. All authors read and approved the final version of the manuscript.

## Conflict of Interest

The authors declare that the research was conducted in the absence of any commercial or financial relationships that could be construed as a potential conflict of interest.
