# Supplementary material for: Reliability, Validity, and Measurement Invariance of the General Anxiety Disorder Scale Among Chinese Medical University Students
Source: Front Psychiatry. 2021 May 19;12:648755. doi: 10.3389/fpsyt.2021.648755 (PMC8170102; doi:10.3389/fpsyt.2021.648755)
Supplement: Supplementary file 1 [file Table_1.DOC]

Supplementary Table 1. χ^2^LD statistic matrix of seven items in the 7-item Generalized Anxiety Disorder Scale

| Item | 1 | 2 | 3 | 4 | 5 | 6 | 7 |
| --- | --- | --- | --- | --- | --- | --- | --- |
| 1 |  |  |  |  |  |  |  |
| 2 | 7.0 |  |  |  |  |  |  |
| 3 | 7.2 | 3.4 |  |  |  |  |  |
| 4 | 4.8 | 3.4 | 9.3 |  |  |  |  |
| 5 | 1.2 | 3.2 | 0.5 | 1.2 |  |  |  |
| 6 | 7.1 | 2.9 | 1.4 | 4.0 | 4.8 |  |  |
| 7 | 0.4 | 2.3 | 1.0 | 2.9 | 3.2 | 2.6 |  |

Supplementary Table 2. Residual covariance matrix of seven items in the 7-item Generalized Anxiety Disorder Scale

| Item | 1 | 2 | 3 | 4 | 5 | 6 | 7 |
| --- | --- | --- | --- | --- | --- | --- | --- |
| 1 |  |  |  |  |  |  |  |
| 2 | 0.028 |  |  |  |  |  |  |
| 3 | -0.007 | -0.004 |  |  |  |  |  |
| 4 | -0.012 | -0.006 | 0.053 |  |  |  |  |
| 5 | 0.005 | 0.005 | -0.013 | -0.018 |  |  |  |
| 6 | -0.007 | -0.011 | -0.005 | -0.005 | 0.004 |  |  |
| 7 | 0.012 | -0.001 | -0.035 | -0.018 | 0.022 | 0.019 |  |

Supplementary Table 3. Conversion list between the original summed GAD score and the IRT trait score by Bayes estimation

| Category | Summed  score | IRT  score | SD | Proportion (%) |
| --- | --- | --- | --- | --- |
| No general anxiety | 0 | -1.77 | 0.51 | 6.90 |
|  | 1 | -1.26 | 0.37 | 7.15 |
|  | 2 | -0.96 | 0.30 | 6.88 |
|  | 3 | -0.71 | 0.26 | 6.30 |
|  | 4 | -0.50 | 0.24 | 6.39 |
| Mild general anxiety | 5 | -0.29 | 0.26 | 7.52 |
|  | 6 | -0.04 | 0.29 | 10.56 |
|  | 7 | 0.25 | 0.30 | 16.19 |
|  | 8 | 0.52 | 0.30 | 9.05 |
|  | 9 | 0.76 | 0.27 | 5.45 |
| Moderate general anxiety | 10 | 0.96 | 0.25 | 3.82 |
|  | 11 | 1.13 | 0.23 | 3.03 |
|  | 12 | 1.29 | 0.23 | 2.59 |
|  | 13 | 1.45 | 0.23 | 2.23 |
|  | 14 | 1.60 | 0.24 | 1.80 |
| Major general anxiety | 15 | 1.75 | 0.24 | 1.29 |
|  | 16 | 1.91 | 0.24 | 0.88 |
|  | 17 | 2.07 | 0.23 | 0.62 |
|  | 18 | 2.24 | 0.24 | 0.46 |
|  | 19 | 2.42 | 0.26 | 0.37 |
|  | 20 | 2.65 | 0.30 | 0.31 |
|  | 21 | 2.96 | 0.39 | 0.22 |

Abbreviation: IRT, item response theory; SD, standard deviation
